# Supplementary material for: Prevalence of fast food consumption and associated factors among secondary school adolescents in Jigjiga Town Somali Region Eastern Ethiopia
Source: PLoS One. 2025 Jul 11;20(7):e0327787. doi: 10.1371/journal.pone.0327787 (PMC12250158; doi:10.1371/journal.pone.0327787)
Supplement: S2 File — Participants information sheet and study questionnaire. (DOCX) [file pone.0327787.s002.docx]

# ANNEX’S

## ANNEX 1: ENGLISH VERSION INFORMATION

**Title of the Project:**

Fast-Food Consumption and Associated Factors among Secondary School Adolescents in Jigjiga Town Somali Region Eastern Ethiopia; 2024.

**The purpose of the study:**

The finding of this study can be of paramount importance for the local & regional decision makers to plan intervention programs to address the problem related to fast food consumption in schools; thereby improve the fast food consumption in general.

**Procedure and duration:**

I am interviewing you using a questionnaire to provide me with pertinent data that is helpful for the study. The interview will take about 15-20 minutes, so I kindly request you to give me your time for the interview.

**Risk and Benefits:**

The risk by being a participant in this study is minimal, but only taking few minutes from your time. There would not be any direct benefits for being participant in this study but the finding from this research may reveal important information for the local planners and decision makers.

**Confidentiality:**

The information you are going to provide us will be kept confidential. In fact, there will not be information that will identify you in particular. The study findings will be general for the study community and will not reflect anything particular to individual persons or housing. The questionnaire will be coded to exclude showing names. There will not be reference in oral or written reports which might link participants to the research.

**Right of the participant:**

Participation in this study is entirely voluntary. You have the right to participate or not, in this study. If you decide to participate, you have the right to withdraw from the study at any time and this will not label you for any loss of benefits to which you are otherwise entitled. You should not have to answer any question you do not want to answer.

## ANNEX’S 2: ENGLISH VERSION QUESTIONNAIRE

| **PART 1:- SOCIO-DEMOGRAPHIC AND ECONOMIC CHARACTERISTICS OF THE RESPONDENTS.** | | | | | | |
| --- | --- | --- | --- | --- | --- | --- |
| **S.No** | **Question for the respondents** | **Response and code** | | | **Remarks** | |
| 01 | What type of school do you attend? | 1. Public school 2. Private school | | |  | |
| 02 | How old are you? | _________ years | | |  | |
| 03 | What is your sex? | 1. Male 2. Female | | |  | |
| 05 | What is your father’s educational level | 1. Unable to read and write 2. Informal education but able to read and or write 3. Primary school level 4. Secondary school level 5. College and above | | |  | |
| 07 | Who are you living with? | 1. Parents 2. Relatives 3. Friends 4. Refuges 5. Alone | | |  | |
| 08 | What are your father’s occupations? | 1. Government employee 2. Private employee 3. Non-governmental(NGO) 4. Merchants 5. Daily labourer 6. Pensioner 7. Others specify __________ | | |  | |
| 09 | Residential area | 1. Urban 2. Ruler | | |  | |
| 10 | What is the Average monthly income of the household? | ___________Birr | | |  | |
| **PART-2:- FAST FOOD CONSUMPTION RELATED QUESSTIONS** | | | | | | |
|  | **Question for the respondents** | | **Response** | | | **Skip** |
| 1 | Do you eat fast foods? (Fried potato products, processed meat products, cakes, sandwich, , Ice cream chocolates, Halawa, Mushebek, soft drinks, burger, pizza, chips, biscuits and etc.) | | 1. Yes 2. No | | |  |
| 2 | How often do you consume fast food in a week? | | 1. More than three days per week 2. Less than three days per week | | |  |
| **PART-3:- FACTORS INFLUENCE FAST FOOD CONSUMPTION RELATED QUESTIONS** | | | | | | |
|  | **Question for the respondents** | **Response** | | | | **Skip** |
| 1 | When do you usually consume fast food during the day? | 1. Break fast 2. Lunch 3. Dinner 4. No specific time | | | |  |
| 2 | Where do you usually consume fast food? | 1. At home 2. At school 3. At friend’s house 4. At restaurant 5. Street food stall | | | |  |
| 3 | Do you have fast foods available at home that can be prepared/eaten instantly? | 1. Yes 2. No | | | |  |
| 4 | Does any fast-food retailers or fast-food restaurant/hotel within the 10 minutes of walking from your home or school? | 1. Yes, I have from home 2. Yes, I have from school 3. Yes, I have from both 4. Not, from both | | | |  |
| 5 | What are the main reasons for consuming fast food? | 1. Taste preference 2. Convenience 3. It is cheaper 4. Easily available at home/school 5. Peer influence 6. Having many variety 7. Others | | | |  |
| 6 | Which is your common source of information about fast foods? | 1. Television advertisement 2. Newspapers, Magazine 3. Outdoor billboards in public places 4. Radio/FM 5. Internet and social network 6. Friends 7. Parents 8. Others specify _______ | | | |  |
| 7 | How much money do you typically spend on fast food per week? | 1. 100-200 birr 2. 300-400 birr 3. ≥ 500 birr | | | |  |
| 8 | How often do you eat fruits and vegetables in a week? | 1. 1–2 times 2. 3–4 times 3. 5 or more | | | |  |
| **PART-4:- KNOWLEDGE RELATED QUESTIONS ABOUT THE HEALTH EFFECT OF FAST FOOD CONSUMPTION.** | | | | | | |
|  | **Question for the respondents** | | | **Response** | | **Remarks** |
| 1 | Bread and baked goods are not good for hypertension patients because they contain high salt levels from the yeast. | | | 1. True 2. False | |  |
| 2 | Fast food such as pizza and fried chick en could cause constipation because of their low vegetable content. | | | 1. True 2. False | |  |
| 3 | Fast food such as hamburgers and pizza contain much fiber which is good for your digestive system. | | | 1. True 2. False | |  |
| 4 | Milk tea such as pearl tea is good for health because it contains both carbohydrate and dairy milk | | | 1. True 2. False | |  |
| 5 | Fried chicken and French fries are filled with trans fats which could cause cardiovascular disease | | | 1. True 2. False | |  |
| 6 | Cola drinks containing high carbohydrates could help digestion | | | 1. True 2. False | |  |
| 7 | Carbohydrate drinks such as Coca Cola, Pepsi and Fanta contain large amounts of sugar which could cause weight gain and obesity. | | | 1. True 2. False | |  |
| 8 | Fast food is typically low in essential nutrients such as vitamins and minerals that are important for overall health. | | | 1. True 2. False | |  |
| 9 | Chronic fast food consumption is associated with an increased risk of obesity and related health problems in the long term. | | | 1. True 2. False | |  |
| `10 | Reducing fast food consumption and adopting a balanced, nutrient-rich diet can have positive effects on overall health and well-being in the long term. | | | 1. True 2. False | |  |

The following questions are about the food situation in your home **during the last month**. Please circle the answer that best describes you.

| **S.No** | **Question for the respondents** | **Response and code** | **Code** |
| --- | --- | --- | --- |
| 1 | Did you **worry** that food at home would run out before your family got money to buy more? | 1. Never 2. Sometimes 3. A lot |  |
| 2 | Did the food that your family bought **run out**, and you didn’t have money to get more? | 1. Never 2. Sometimes 3. A lot |  |
| 3 | Did your meals only include a few kinds of **cheap foods** because your family was running out of money to buy food? | 1. Never 2. Sometimes 3. A lot |  |
| 4 | How often were you not able to eat a **balanced meal** because your family didn’t have enough money? | 1. Never 2. Sometimes 3. A lot |  |
| 5 | Did you have to **eat less** because your family didn’t have enough money to buy food? | 1. Never 2. Sometimes 3. A lot |  |
| 6 | Has the size of your meals **been cut** because your family didn’t have enough money for food? | 1. Never 2. Sometimes 3. A lot |  |
| 7 | Did you have to **skip a meal** because your family didn’t have enough money for food? | 1. Never 2. Sometimes 3. A lot |  |
| 8 | Were you **hungry** but didn’t eat because your family didn’t have enough food? | 1. Never 2. Sometimes 3. A lot |  |
| 9 | Did you not eat for a **whole day** because your family didn’t have enough money for food? | 1. Never 2. Sometimes 3. A lot |  |
