# Supplementary material for: Prevalence of fast food consumption and associated factors among secondary school adolescents in Jigjiga Town Somali Region Eastern Ethiopia
Source: PLoS One. 2025 Jul 11;20(7):e0327787. doi: 10.1371/journal.pone.0327787 (PMC12250158; doi:10.1371/journal.pone.0327787)
Supplement: Attachment — Submitted filename: Response_to_Reviewers_auresp_2.docx [file pone.0327787.s006.docx]

**Response to the Reviewers**

PONE-D-25-10434R1

PREVALENCE OF FAST FOOD CONSUMPTION AND ASSOCIATED FACTORS AMONG SECONDARY SCHOOL ADOLESCENTS IN JIGJIGA TOWN SOMALI REGION EASTERN ETHIOPIA
**ACADEMIC EDITOR Comments:** - The manuscript still requires language and grammatical revision. - The rationale for selecting in the multivariate model (p < 0.25) needs to be justified with proper references. - Enhancing the cultural context in your discussion—especially regarding gender differences and socioeconomic influences—would strengthen the interpretive depth of the findings.

**Response to Academic Editor:**

Dear Editor, Thank you for your valuable comments and thoughtful suggestions. We would like to indicate that all the comments have been carefully corrected and addressed as follows:

- The manuscript has undergone thorough language and grammatical revision to enhance clarity and readability.
- The rationale for selecting variables with a p-value < 0.25 in the multivariate model has been clearly justified and supported with proper reference in the revised manuscript.
- The discussion section has been expanded to better reflect the cultural context, particularly in relation to gender differences and socio-economic influences on fast food consumption, to provide deeper interpretive insight in to the findings.

**Reviewers' comments:**

**Reviewer #1:**

NONE, it can be accepted. All the comments replied and included in the text.
It was my previous comments to author.

It needs a strong revision, as there are serious readability problems and methodological issues that the author(s) must manage effectively. All Parts of manuscript need revision. Actually, I suggest the authors study more articles in order to know the method of article writing. For example, it is about strength of statistical analysis in abstract that is unusual. The aim of introduction is not complete. There are lots of sentences that are vague and incomplete. Therefore, it is important to review the article’ writing. There are some typos throughout the text. The reference part is not regarding with Guideline of journal. The quality of figures is so weak and the data is presented in content of manuscript. The format of section Conclusion is like a report not article.

**Response to Reviewer #1:**

**Dear Reviewer,**

We would like to express our sincere appreciation for the focused and constructive comments provided on our manuscript.

We would like to note that most of the issues raised were addressed in the previous revision, as acknowledged in the reviewer’s initial statement: "NONE, it can be accepted. All the comments replied and included in the text. It was my previous comments to author."

However, in light of the additional comments provided, we have carefully reviewed the manuscript once again and have made further revisions to ensure the quality and clarity of the work. Specifically:

- **Readability and Structure:** The manuscript has undergone a comprehensive language and grammar review to enhance clarity, coherence, and flow throughout all sections.
- **Conclusion Section:** The conclusion has been rewritten to better reflect an academic tone, focusing on summarizing key findings and implications, rather than reporting.

**Reviewer #2:**

**Dear Authors,** This study presents valuable insights into the prevalence and determinants of fast food consumption among adolescents in Jigjiga, an under-researched region in Eastern Ethiopia. The topic is both timely and relevant to global public health discourse, particularly in low- and middle-income contexts. Your revisions demonstrate responsiveness to earlier feedback, especially with the expanded rationale, improved methodology, and inclusion of ethical and statistical clarifications. However, the manuscript still requires significant refinement in scientific language and consistency. Several sections, particularly the Abstract, Discussion, and Conclusion, contain grammatical errors, redundant phrasing, and lack the academic tone expected in peer-reviewed journals. Furthermore, operational definitions should be presented clearly and consistently, and the rationale for variable inclusion in the multivariate model (p < 0.25) needs to be justified with proper references. Enhancing the cultural context in your discussion—especially regarding gender differences and socioeconomic influences—would strengthen the interpretive depth of the findings.

**Response to Reviewer #2:**

**Dear Reviewer,**

Thank you very much for your thoughtful and constructive comments and suggestions, as well as for acknowledging the relevance and improvements made in our manuscript. We greatly appreciate your insights, which have helped us further improve the quality of this manuscript.

In response to your valuable comments and suggestions, we have made the following revisions:

- The **Abstract, Discussion, and Conclusion** was edited to enhance scientific language, remove redundancies, and ensure a consistent academic tone.
- We revised the **operational definitions** for clarity and consistency.
- We incorporated a **justification with appropriate references** for including variables with p < 0.25 in the multivariate model.
- The discussion is strengthened with the **cultural context** in the discussion, particularly regarding **gender differences** and **socioeconomic influences.**
